# Safety and efficacy of robot-assisted bile ductoplasty and intrapancreatic bile duct resection in congenital biliary dilatation: a single-center retrospective cohort (2013–2024)

**DOI:** 10.1007/s11701-025-02782-8

**Published:** 2025-09-18

**Authors:** Daiki Kato, Chiyoe Shirota, Hiroo Uchida, Akinari Hinoki, Satoshi Makita, Katsuhiro Ogawa, Masamune Okamoto, Akihiro Yasui, Shunya Takada, Kaito Hayashi, Yoichi Nakagawa, Hiroki Ishii, Hajime Asai, Hizuru Amano, Takahisa Tainaka

**Affiliations:** Department of Pediatric Surgery, Nagoya University Graduate School of Medicine, 65 Tsurumai-cho, Showa-ku, Nagoya, 466-8550 Japan

**Keywords:** Robot-assisted surgery, Congenital biliary dilatation, Ductoplasty, Intrapancreatic bile duct resection

## Abstract

Robot-assisted surgery is increasingly being utilized for congenital biliary dilatation (CBD); however, limited data exist on its feasibility across age groups, particularly for bile ductoplasty and intrapancreatic bile duct (IPBD) resection. This was a retrospective cohort study that compared the outcomes of robot-assisted versus laparoscopic CBD surgery in 159 patients (Rob group: 57; Lap group: 102) treated between 2013 and 2024. In the Rob group, hospital stay was significantly shorter, enteral feeding was started earlier, and drain duration was reduced, especially in pediatric patients. Late complications (e.g., cholangitis) were significantly less common in the Rob group compared to the Lap group (3.5 vs. 18%). The length of residual IPBD was similar in both groups. Furthermore, robotic procedures facilitate a secure and accurate hilar bile ductoplasty, thereby contributing to favorable outcomes without anastomotic stenosis or hepatolithiasis. While these findings support the potential advantages of robot-assisted CBD surgery, the follow-up period was significantly shorter in the Rob group than in the Lap group, and thus definitive conclusions regarding long-term outcomes cannot yet be drawn. Robot-assisted surgery appears safe and is associated with faster short-term recovery; long-term comparative effectiveness requires longer follow-up and adjusted analyses.

**Supplementary Information:**

The online version contains supplementary material available at 10.1007/s11701-025-02782-8.

## Introduction

Postoperative complications of congenital biliary dilatation (CBD) include hepatolithiasis, cholangitis, cholangiocarcinoma, pancreatitis, and pancreatolithiasis [1–4]. Congenital intrahepatic bile duct (IHBD) and anastomotic stenoses are recognized as the major causes of hepatolithiasis and cholangitis after CBD surgery [1]. In addition, the residual intrapancreatic bile duct (IPBD) is linked to the development of carcinoma, pancreatitis, and pancreatolithiasis [5]. To prevent these postoperative complications, we adopted a surgical strategy that included aggressive bile ductoplasty for the IHBD stenosis and complete resection of the IPBD, both open and laparoscopic surgeries, respectively.

In recent years, robot-assisted surgery has been increasingly applied to CBD, and several studies have compared its outcomes with those of laparoscopic surgery for CBD [6]. However, few reports have described the technical feasibility of bile ductoplasty and IPBD resection using robot-assisted surgery. Moreover, most previous studies have focused exclusively on pediatric or adult patients. As far as we know, no previous study has evaluated the efficacy of CBD surgery with robot-assisted surgery across a broad age and weight spectra, including neonates as small as weighing 3 kg and adults up to 90 kg and 60 years of age.

This study aimed to investigate whether bile ductoplasty and complete resection of IPBD using robot-assisted surgery are as safe and effective as those performed via laparoscopic surgery in this diverse patient population.

## Methods

Approval to conduct this study was obtained from the Ethical Review Committee of Nagoya University Hospital (2025–177) and conformed to the 1964 Declaration of Helsinki. Details of all study protocols are available on our institutional website. Informed consent was obtained through an opt-out method after IRB approval.

The study retrospectively analyzed data from CBD patients who underwent robot-assisted or laparoscopic surgery, including complete cyst excision, hepaticojejunostomy, and Roux-en-Y limb reconstruction, at our institution between 2013 and 2024. Non-dilation type of Todani classification cases were defined as patients who underwent radical surgery for pancreaticobiliary maljunction without congenital biliary dilatation.

The patients were divided into two groups: the robot-assisted (Rob group) and laparoscopic (Lap group) surgery groups. Patient data and outcomes were compared between the groups, including subgroup analyses for children and adults (defined as ≥ 18 years). Furthermore, surgical outcomes were compared between cases in which bile ductoplasty was performed and those in which it was not. Laparoscopic surgery was initiated in 2013, and robot-assisted surgery in 2021 at our institution. Both procedures were performed during the period from 2021 to 2023.

The laparoscopic surgical and robot-assisted procedures used at our hospital have been described previously [7, 8]. The details of the procedure are described in the supplementary information (SI. 1 and 2). Supplementary Fig. 1 shows the port maps.

Membrane or septal bile duct stricture was routinely resected. If a structure was identified from the hepatic hilum to the secondary branches, it was routinely resected, and the cut surface was sutured using 5-0 or 6-0 absorbable sutures to achieve hemostasis and prevent cicatricial stricture. This procedure is known as bile ductoplasty. When necessary, such as when the stenosis was deep or complex in form, intraoperative cholangiography was performed.

We describe the creation of a Roux-en-Y limb. The small intestine was exteriorized through an umbilical incision in all patients. The limb was constructed extracorporeally and transported to the hepatic hilum. In pediatric patients, the retrocolic route was prepared extracorporeally, whereas in adult patients, the retrocolic route and limb positioning were performed intraperitoneally. The mesenteric defect and Petersen’s space were routinely closed to prevent internal herniation. The choice of approach (extracorporeal or intraperitoneal) was determined intraoperatively based on the patient’s size and anatomy.

There were no strictly standardized perioperative protocols, and no significant changes were observed during the study period (2013–2024). There was no regular administration of analgesics, and acetaminophen (10–15 mg/kg) was administered as needed. After confirming no abnormal findings on abdominal X-ray images and good intestinal motility, fluid intake was initiated, and if there were no issues with fluid intake, food intake was started. Regarding drains, they were removed after confirming that the drainage volume was low and there were no abnormalities in the drainage characteristics.

Postoperative evaluation included blood tests, ultrasonography, magnetic resonance cholangiopancreatography (MRCP), or computed tomography. If there are no postoperative complications or symptoms, MRCP is performed at 1, 3, 5, 7, 10, 15, and 20 years after surgery to check for IHBD stenosis or residual IPBD. If complications occur or symptoms appear, additional blood tests and imaging tests are performed as appropriate. The presence or absence of residual IPBD and their length are measured by pediatric surgeons using the MRCP scaling function, and consensus is reached at conferences.

Early complications were defined as ≤ 30 days postop and categorized by Clavien–Dindo; late complications were > 30 days and analyzed both as any grade and as ≥ IIIa. Cholangitis was defined per Tokyo Guidelines 2018 [9].

In our hospital, double-balloon endoscopic retrograde cholangiography has been the first-line diagnostic and therapeutic tool for IHBD stones and cholangitis since 2013. We diagnosed hepaticojejunostomy anastomotic stenosis when no biliary mucosa was visible between the stenosis and the anastomosis. On the other hand, when the mucosa was visible, we diagnosed IHBD stenosis.

We present continuous variables as medians and quartile ranges, and categorical variables as frequencies and percentages. We used Fisher’s exact test for categorical data comparisons and applied the Mann–Whitney *U* test for continuous variable comparisons. We constructed multivariable regression models (logistic and Cox) adjusted for age, weight, Todani type, perforation at presentation, approach (laparoscopy vs robot assisted), and performance of bile ductoplasty. Considering the evolution of perioperative care, imaging diagnostics, and surgeon experience over time, sensitivity analysis was performed exclusively on cases from 2021 to 2023 (the period when both surgical approaches coexisted). Time-to-event outcomes (first late complication) were analyzed with Kaplan–Meier models. We report effect sizes with 95% confidence intervals (CI) as for key results. A *p* value less than 0.05 was considered statistically significant. This study is exploratory in nature and aims to generate hypotheses, so multiple comparison *p* value correction was not performed. Individual *p* values are presented as a reference for hypothesis verification.

## Results

**Fig. 1:**
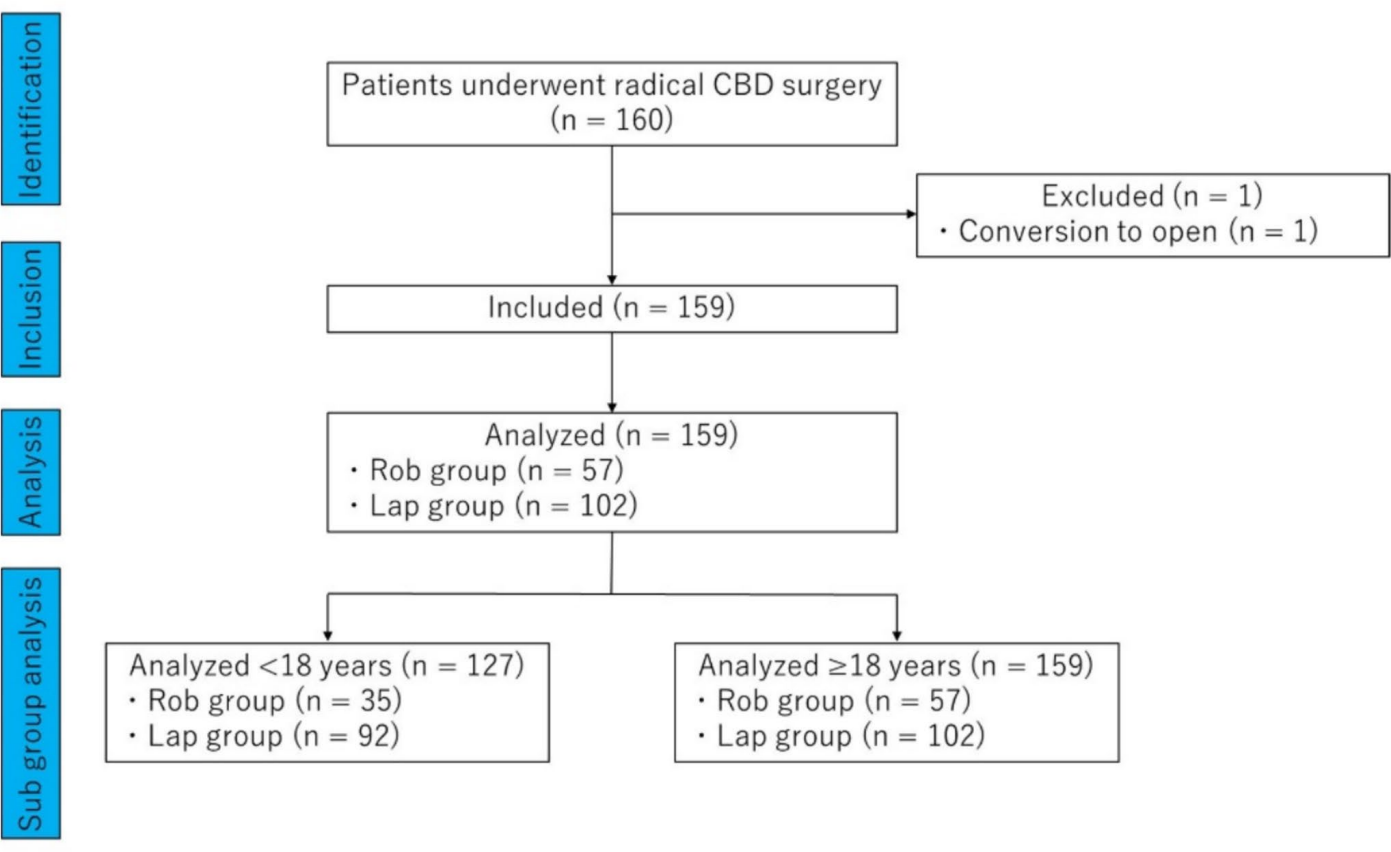
STROBE-compliant flow diagram

A total of 160 patients underwent radical CBD surgery. Cases involving palliative surgery such as biliary drainage and reoperation were not included. In the Rob group, the surgery was converted to open in a 16-year-old patient because of an anatomic abnormality involving a duodenal diverticulum perforating into the common bile duct: this case was excluded from the analysis. There were no cases of conversion to open surgery in the Lap group. A total of 159 patients were included in this study (Rob group: 57, Lap group: 102). Figure 1 shows a diagram of this study.

Table 1 summarizes patient characteristics. The Rob group had significantly higher age and body weight at the time of surgery (median age: 9 vs. 3 years; weight: 29 vs. 13 kg; both *p* < 0.001). Compared with that at the beginning of 2013, adult CBD cases at our institution have gradually increased. The follow-up period was significantly shorter in the Rob group (1 vs. 6 years, *p* < 0.001), reflecting the more recent adoption of robotic surgery.

**Table 1:** Characteristics of the patients who underwent radical surgery for congenital biliary dilatation

	Rob (*n* = 57)	Lap (*n* = 102)	*p* value
Male	20 (35%)	26 (25%)	0.21
Age (years)	9 (2–24)	3 (1–8)	** < 0.001**
< 1 year	7 (12%)	21 (21%)	
1–17 years	28 (49%)	71 (70%)	
≥ 18 years	22 (39%)	10 (9.8%)	
Body weight (kg)	29 (14–55)	13 (9–23)	** < 0.001**
Todani classification			0.12
1a	14	17	
1b	1	0	
1c	12	14	
IV-A	26	66	
Non-dilatation	4	5	
Perforation	1 (1.8%)	4 (3.9%)	0.66
Follow-up period (years)	1 (1–2)	6 (5–8)	** < 0.001**

Values are present as *n* (%) or median (interquartile range). Bold value indicates significant difference

*p* values are two-sided

*Rob* patients underwent robotic-assisted surgery; *Lap* patients underwent laparoscopic surgery

The surgical outcomes are summarized in Table 2. The operative time of the Rob group included the time required for docking the robot. The operative time was significantly longer (454 vs. 408 min, *p* = 0.013), but the length of hospital stay (8 vs. 10 days, *p* < 0.001), time to enteral feeding (3 vs. 4 days, *p* = 0.001), and duration of drain placement (5 vs. 6 days, *p* = 0.002) were significantly shorter in the Rob group than in the Lap group. Early complications rate was comparable (12% vs. 7.8%, *p* = 0.40); however, late complications (Clavien–Dindo classification I or higher) occurred significantly more often in the Lap group (3.5% vs. 18%, *p* = 0.011), particularly cholangitis (*p* = 0.033). The length of residual IPBD was similar in both groups. No cholangiocarcinomas were observed.

**Table 2:** Surgical outcomes of the patients who underwent radical surgery for congenital biliary dilatation

	Rob (*n* = 57)	Lap (*n* = 102)	*p* value
Operative time (min)	454 (368–556)	408 (352–455)	**0.013**
Blood loss (mL)^a^	27 (17–46)	38 (12–82)	0.17
Bile ductoplasty	29 (51%)	58 (57%)	0.51
Early complication^b^	7 (12%)	8 (7.8%)	0.40
Bile leakage	3^e^	6	0.76
Pancreatic fistula	3^e^	2	0.29
Bowel obstruction	2	0	0.20
Roux-en-Y limb dilatation	1	0	0.72
Length of hospital stay (days)	8 (7–11)	10 (8–13)	** < 0.001**
Period until enteral feeding starts (days)	3 (3–4)	4 (3–5)	**0.001**
Duration of drain placement (days)	5 (5–7)	6 (6–7)	**0.002**
Late complication (CD ≥ I)^c^	2 (3.5%)	18 (18%)	**0.011**
Anastomotic stenosis	0	3 ^f^	0.55
Hepatolithiasis	0	7 ^f^	0.050
Cholangitis	1	12 ^f^	**0.033**
Pancreatolithiasis	0	1	1
Pancreatitis	0	1	1
Bowel obstruction	1	1	1
Late complication (CD ≥ III)^d^	1 (1.8%)	12 (12%)	**0.033**
Anastomotic stenosis	0	3^ g^	0.55
Hepatolithiasis	0	6^ g^	0.089
Cholangitis	0	6^ g^	0.089
Pancreatolithiasis	0	1	1
Bowel obstruction	1	1	1
Residual intrapancreatic bile duct (mm)	0 (0–5)	0 (0–0)	0.13
Cholangiocarcinoma	0	0	

Values are present as *n* (%) or median (interquartile range). Bold value indicates significant difference

*p* values are two-sided

*Rob* pPatients underwent robotic-assisted surgery; *Lap* patients underwent laparoscopic surgery

a: Blood loss was measured intraoperatively

b: Complications within 30 days after surgery and Clavien–Dindo classification III or higher

c: Complications 31 days or more after surgery and Clavien–Dindo classification I or higher including events treated conservatively

d: Complications 31 days or more after surgery and Clavien–Dindo classification III or higher including events treated conservatively

e: Ttwo patients developed bile leakage and pancreatic fistula

f: Five patients developed hepatolithiasis and cholangitis, and one patient developed hepatolithiasis and anastomotic stenosis

g: Five patients developed hepatolithiasis and cholangitis, and one patient developed hepatolithiasis and anastomotic stenosis

Supplementary Fig. 2 shows the Kaplan–Meier curve representing the time to onset of long-term complications. There was no significant difference in late complication-free survival between the two groups. At 1 year postoperatively, the complication-free survival rate and 95% CI were 92% (85–96) and 98% (88–100) in the Lap and Rob groups, respectively.

The pediatric outcomes (< 18 years) are summarized in Table 3. The Rob group had significantly shorter hospital stay (7 vs. 10 days, *p* < 0.001), earlier enteral feeding (3 vs. 4 days, *p* < 0.001), and shorter drain duration (5 vs. 6 days, *p* < 0.001) than the Lap group. The incidence of late complications (Clavien–Dindo classification I or higher) tended to be higher in the Lap group (2.9% vs. 16%, *p* = 0.068). No residual IPBD or cholangiocarcinoma was observed in this age group.

**Table 3:** Surgical outcomes, < 18 years

	Rob (*n* = 35)	Lap (*n* = 92)	*p* value
Operative time (min)	388 (345–521)	395 (343–450)	0.55
Blood loss (mL)^a^	22 (15–36)	37 (12–74)	0.063
Bile ductoplasty	17 (49%)	54 (59%)	0.32
Early complication^b^	4 (11%)	6 (6.5%)	0.46
Bile leakage	1	5	0.33
Pancreatic fistula	1	1	0.46
Bowel obstruction	2	0	0.26
Length of hospital stay (days)	7 (7–9)	10 (8–13)	** < 0.001**
Period until enteral feeding starts (days)	3 (3–4)	4 (3–5)	** < 0.001**
Duration of drain placement (days)	5 (5–6)	6 (6–7)	** < 0.001**
Late complication (CD ≥ I)^c^	1 (2.9%)	15 (16%)	0.068
Anastomotic stenosis	0	3^e^	0.56
Hepatolithiasis	0	5^e^	0.32
Cholangitis	1	8^e^	0.28
Pancreatolithiasis	0	1	1
Pancreatitis	0	1	1
Bowel obstruction	0	1	1
Late complication (CD ≥ III)^d^	0	9	0.062
Anastomotic stenosis	0	3^f^	0.56
Hepatolithiasis	0	5^f^	0.32
Cholangitis	0	3^f^	0.56
Pancreatolithiasis	0	1	1
Bowel obstruction	0	1	1
Residual intrapancreatic bile duct (> 10 mm)	0	0	
Cholangiocarcinoma	0	0	

Values are present as *n* (%) or median (interquartile range). Bold value indicates significant difference

*p* values are two-sided

*Rob* patients underwent robotic-assisted surgery; *Lap* patients underwent laparoscopic surgery

a: Blood loss was measured intraoperatively

b: Complications within 30 days after surgery and Clavien–Dindo classification III or higher

c: Complications 31 days or more after surgery and Clavien–Dindo classification I or higher including events treated conservatively

d: Complications 31 days or more after surgery and Clavien–Dindo classification III or higher including events treated conservatively

e: Three patients developed hepatolithiasis and cholangitis, and one patient developed hepatolithiasis and anastomotic stenosis

f: Three patients developed hepatolithiasis and cholangitis, and one patient developed hepatolithiasis and anastomotic stenosis

The adult outcomes (≥ 18 years) are summarized in Table 4. Operative time was comparable (520 vs. 519 min, *p* = 0.98), but blood loss tended to be lower in the Rob group than in the Lap group (35 vs. 81 mL, *p* = 0.059). The early complication rates were similar (14 vs. 20%, *p* = 0.64). Late complication (Clavien–Dindo classification I or higher) rates and residual IPBD incidence showed no significant differences; however, cholangitis occurred significantly more often in the Lap group than in the Rob group (30 vs. 0%, *p* = 0.024). No cholangiocarcinomas were observed in either group. Supplementary Fig. 3 shows the Kaplan–Meier curve representing the time to onset of long-term complications in adult patients. At 1 year postoperatively, the complication-free survival rate was significantly higher in the Rob group (96 vs. 70%, CI 72–93 vs. 33–89, *p* = 0.046).

**Table 4:** Surgical outcomes, ≥ 18 years

	Rob (*n* = 22)	Lap (*n* = 10)	*p* value
Operative time (min)	520 (464–581)	519 (444–649)	0.98
Blood loss (mL)^a^	35 (24–89)	81 (60–353)	0.059
Bile ductoplasty	12 (55%)	4 (40%)	0.70
Early complication^b^	3 (14%)	2 (20%)	0.64
Bile leakage	2^d^	1	1
Pancreatic fistula	2^d^	1	1
Roux-en-Y limb dilatation	1	0	1
Length of hospital stay (days)	8 (7–13)	13 (9–15)	0.15
Period until enteral feeding starts (days)	3 (3–5)	4 (3–5)	0.98
Duration of drain placement (days)	6 (5–9)	6 (6–8)	0.85
Late complication (CD ≥ I)^c^	1 (4.5%)	3 (30%)	0.079
Hepatolithiasis	0	2^e^	0.091
Cholangitis	0	3^e^	**0.024**
Bowel obstruction	1	0	1
Late complication (CD ≥ III)^d^	1 (4.5%)	3 (30%)	0.079
Hepatolithiasis	0	2^f^	0.091
Cholangitis	0	3^f^	**0.024**
Bowel obstruction	1	0	1
Residual intrapancreatic bile duct (> 10 mm)	2 (9.1%)	1 (10%)	1
Cholangiocarcinoma	0	0	

Values are present as *n* (%) or median (interquartile range). Bold value indicates significant difference

*p* values are two-sided

*Rob* patients underwent robotic-assisted surgery; *Lap* patients underwent laparoscopic surgery

a: Blood loss was measured intraoperatively

b: Complications within 30 days after surgery and Clavien–Dindo classification III or higher

c: Complications 31 days or more after surgery and Clavien–Dindo classification I or higher including events treated conservatively

d: Two patients developed bile leakage and pancreatic fistula

e: Two patients developed hepatolithiasis and cholangitis

f: Two patients developed hepatolithiasis and cholangitis

The comparison of surgical outcomes between the group that underwent bile ductoplasty and the group that did not is shown in Supplementary Table 1. Operative time was longer in the bile ductoplasty + group than in the bile ductoplasty group—(*p* < 0.05). There was no difference in the incidence of early complications, hepatolithiasis, or cholangitis.

Logistic regression analysis revealed that robot-assisted surgery (OR 0.115, 95% CI 0.022–0.594; *p* = 0.010) was significantly associated with the decrease in the incidence of late complications (Supplementary Table 2).

The difference in median length of hospital stay between the Rob group and the Lap group was 2 days, and the difference in the time to initiation of enteral feeding and the duration of drain placement was 1 day. Cox regression analysis showed that robot-assisted surgery remained an independent predictor for a shorter length of hospital stay (HR 1.680, 95% CI 1.154–2.446; *p* = 0.018), time to enteral feeding (HR 1.602, 95% CI 1.106–2.322; *p* = 0.013), and duration of drain placement (HR 1.560, 95% CI 1.062–2.291; *p* = 0.023) (Supplementary Table 3–5).

Table 5 shows the results of a sensitivity analysis for the period from 2021 to 2023. There were no significant differences between the two groups in age, weight, duration until nutritional initiation, duration of drain placement, or complication rates. The length of hospital stay was significantly shorter in the Rob group.

**Table 5:** Characteristics and outcomes of patients who underwent radical surgery between 2021 and 2023

	Rob (*n* = 43)	Lap (*n* = 9)	*p* value
Male	17 (40%)	5 (30%)	0.47
Age (years)	6 (2–24)	5 (1–9)	0.41
< 1 year	6 (14%)	2 (22%)	
1–17 years	23 (53%)	5 (56%)	
≥ 18 years	14 (33%)	2 (22%)	
Body weight (kg)	22 (11–51)	15 (11–34)	0.38
Todani classification			0.44
1a	10 (23%)	1 (11%)	
1b	1 (2.3%)	0	
1c	7 (16%)	0	
IV-A	21 (49%)	8 (89%)	
Non dilataion	4	0	
Perforation	0	1 (11%)	0.17
Operative time (min)	454 (375–559)	453 (410–513)	0.66
Blood loss (mL)^a^	27 (17–42)	47 (12–371)	0.35
Bile ductoplasty	20 (47%)	6 (67%)	0.47
Early complication^b^	5 (12%)	2 (22%)	0.59
Length of hospital stay (days)	8 (7–9)	12 (9–14)	**0.006**
Period until enteral feeding starts (days)	3 (3–4)	4 (3–5)	0.11
Duration of drain placement (days)	5 (5–6)	6 (6–10)	0.063
Late complication^c^	2 (4.7%)	0	1
Bowel obstruction	1	0	1
Cholangitis	1	0	1
Residual intrapancreatic bile duct (mm)	0 (0–5)	0 (0–0)	0.37
Cholangiocarcinoma	0	0	

Values are present as *n* (%) or median (interquartile range). Bold value indicates significant difference

*p* values are two-sided

*Rob* patients underwent robotic-assisted surgery; *Lap* patients underwent laparoscopic surgery

a: Blood loss was measured intraoperatively

b: Complications within 30 days after surgery and Clavien–Dindo classification III or higher

c: Complications 31 days or more after surgery and Clavien–Dindo classification I or higher including events treated conservatively

A sensitivity analysis was performed excluding cases of non-dilation type (*n* = 9) (Supplementary Table 6). In this subgroup analysis, the results were similar to those of the overall analysis.

## Discussion

In this study, robot-assisted surgery demonstrated safety equivalent to laparoscopic surgery, with no significant difference in early complication rates. Despite a shorter follow-up period, robot-assisted surgery was associated with accelerated postoperative recovery, particularly in pediatric patients, and showed the potential to reduce long-term postoperative complications.

Complications after CBD surgery can occur on both the hepatic and pancreatic sides. On the hepatic side, IHBD stenosis is a known risk factor for hepatolithiasis, cholangitis, and cholangiocarcinoma. Most IHBD stenoses in the CBD are considered congenital, resulting from septal or membranous strictures [7]. Hepatolithiasis can lead to recurrent cholangitis, a major risk factor for bile duct cancer [1, 2].

To mitigate these risks, we routinely performed bile ductoplasty for hilar IHBD stenosis in both open and laparoscopic surgery, and favorable outcomes of hilar bile ductoplasty have been previously reported [7]. Careful examination and removal of the membrane or septum that causes IHBD stenosis have been shown to effectively prevent hepatolithiasis after surgery. In this study, we performed bile ductoplasty in more than 50% of robot-assisted surgeries. These procedures increased the operative time, but did not increase complications and demonstrated feasibility comparable to that of laparoscopic procedures. The robotic platform offers advantages, such as tremor filtration, motion scaling, and three-dimensional visualization, which enable more precise and thorough dissection. In this study, the robot’s 3D magnification and multi-joint functionality enabled relatively easy and safe bile ductoplasty for incision and suturing of a stricture area that would be difficult to access laparoscopically (located deep in the hepatic hilum, with the direction of the operation on the stricture area not matching the axis direction of the forceps) (Supplementary Fig. 4). Several studies reported that robot-assisted surgery was more effective than laparoscopic surgery, particularly in terms of anastomosis and suturing [10, 11]. Although bile ductoplasty in robot-assisted surgery has not been widely evaluated, our findings suggest that the system facilitates accurate handling of stenotic lesions. In the Rob group, no anastomotic stenosis and only one case of hepatolithiasis/cholangitis were observed, despite the shorter follow-up period.

However, certain peripheral IHBD stenoses, particularly those located deep within the liver, cannot be surgically addressed. Stenosis in the hilar region or near the secondary branches can be removed using intraoperative bile ductoplasty. In one patient in the Rob group, cholangitis developed postoperatively due to pre-existing stenosis in the right posterior section, which could not be surgically corrected. These deep IHBD anomalies are rare but warrant long-term follow-up. Intraoperative bile ductoplasty remains important in reducing the risk of complications.

In the pancreas, residual IPBD can cause postoperative pancreatitis, pancreatolithiasis, and carcinoma [12]. Therefore, complete IPBD excision should be performed. As far as we know, no reports have evaluated robot-assisted surgery for the CBD in detail with respect to intrapancreatic ductal treatment. In this study, no significant difference in the incidence of residual IPBD or related complications was observed between groups. In CBD surgery, we perform intraoperative cholangiography to evaluate the anatomy of the bile duct and pancreatic duct, and then resect the intrapancreatic bile duct at the very edge of the pancreatic duct junction. The detachment of the IPBD requires extremely delicate manipulation to avoid damaging the blood vessels and pancreatic tissue surrounding the bile duct. Robot-assisted surgery provides a high-definition 3D view and magnified field of view, enabling clearer visualization of fine blood vessels and the boundaries between pancreatic tissue and bile ducts. This allows safe and accurate dissection. In addition, multi-jointed forceps and image stabilization enable manipulation in deep, narrow spaces that are difficult to access with laparoscopic surgery, allowing for more precise and stable incisions and dissections.

In the Rob group, hospital stay was significantly shorter, enteral feeding was started earlier, and drain duration was reduced. These benefits were even more pronounced in pediatric patients. International multicenter studies and meta-analyses have also demonstrated the shorter postoperative hospital stay or fewer postoperative complications associated with robot-assisted surgery, demonstrating its safety and feasibility [6, 13, 14]. These findings suggest that robot-assisted surgery may reduce surgical trauma, minimize bowel manipulation, and expedite the recovery of intestinal function.

This study included a wide patient population ranging from neonates weighing approximately 3 kg to adult patients weighing up to 90 kg aged 60 years. This diversity not only adds to the generalizability of the study but also raises important questions regarding patient selection, surgical adaptation, and perioperative management across age groups. It is especially remarkable that robot-assisted surgery demonstrated comparable or even superior short-term outcomes, such as shorter hospital stay and faster initiation of enteral feeding, despite the inclusion of adult cases. Furthermore, the subgroup analysis suggested that robot-assisted surgery accelerates postoperative recovery, especially in pediatric patients. Minimally invasive surgery is particularly important in children, and these results support the widespread use of robot-assisted surgery in pediatric surgery.

The operative time was significantly longer in the Rob group than in the Lap group, likely due to the greater proportion of adult cases. Adult patients tend to have more severe inflammation and adhesions owing to delayed diagnosis. In addition, in adult cases, the Roux-en-Y limb was constructed intraperitoneally and routed via the retrocolic pathway, with intraperitoneal fixation of the limb and closure of the mesenteric defect and Petersen’s space. These technical steps, which are not typically required in pediatric cases, may have contributed to longer operative times. Nonetheless, subgroup analyses showed comparable operative durations between the Rob and Lap groups when stratified by age, suggesting that these differences were due to patient background and procedural complexity rather than the robotic approach itself. Moreover, previous studies indicated that although the total operation time was longer, robot-assisted surgery resulted in significantly shorter cyst resection and hepaticojejunostomy times [15]. There are reports that robot-assisted surgery is useful in reducing the time required for hepaticojejunostomy compared to laparoscopic surgery, not only in children but also in adults [16, 17]. The long duration of hepaticojejunostomy in robot-assisted surgery was thought to be due to the time required for docking and the time needed to change the equipment. It was suggested that the longer surgery times in the Rob group were due to the higher number of adult cases, and once the surgeon overcomes the learning curve, surgery time would be further reduced.

Laparoscopic surgery was performed from 2013, while robot-assisted surgery began in 2021, reflecting changes in surgical techniques over time. Analysis limited to the period when both procedures were performed (2021–2023) showed that the significant difference in enteral feeding initiation period and drain placement period disappeared; however, a significant difference in length of hospital stay remained. These results suggest that advances in perioperative care, imaging, and surgeon experience may have improved overall outcomes in the Rob group, demonstrating superiority in comparisons across all periods. Logistic regression analysis demonstrated that robot-assisted surgery was an independent predictor significantly reducing the risk of complications. Furthermore, Cox regression analysis demonstrated that robot-assisted surgery was an independent predictor for shorter hospital stays, earlier initiation of feeding, and shorter drain retention periods. After adjusting for era effects and patient background, robot-assisted surgery was suggested to potentially yield improvements in postoperative outcomes. Therefore, this study suggested that robot-assisted surgery may have an advantage over laparoscopic surgery in accelerating postoperative recovery.

In view of the fact that cases of non-dilatation type were included in the cohort, which could act as a confounding factor, a sensitivity analysis was conducted. Sensitivity analysis showed that excluding these cases did not significantly alter our main findings. These results indicate that the presence or absence of bile duct dilatation did not significantly affect the conclusions of this study, reinforcing the robustness of our main findings. However, for a more detailed analysis, large-scale studies involving a large number of cases of non-dilatation type will be necessary in the future.

We describe some limitations. First, this was retrospective study. However, our center has performed the largest number of robot-assisted CBD surgeries in Japan; selection bias may have been introduced, especially as more adult cases were included in the Rob group due to the recent expansion of robot-assisted surgery. The significantly high proportion of adult patients in the robot group may still cause bias in the results. We did not intentionally choose laparoscopic or robot-assisted surgery based on the difficulty of the cases. Laparoscopic and robot-assisted surgeries were performed in patients requiring emergency surgery due to bile duct perforation. Second, the shorter follow-up period in the Rob group (median 1 vs. 6 years in the Lap group) may underestimate the incidence of late complications. The results of this study are valid for comparing short-term postoperative outcomes, but conclusions regarding long-term late complications should be interpreted with caution. Our findings should be validated in long-term prospective multicenter studies with larger cohorts. Third, based on the status of robot introduction in 2021, the results may reflect initial experiences. Previous studies have reported that approximately 25–30 cases are required for the learning curve of robot-assisted CBD surgery to stabilize [10, 18]. During the study period, we were in the middle of the learning curve. At this stage, there may be variability in surgical time and some postoperative outcomes. While it cannot be ruled out that this may have influenced the results of this study, it is considered that it may provide important information for evaluating outcomes after CBD surgery. Fourth, this study did not perform multiple comparison corrections. The possibility of statistical significance being obtained by chance could not be ruled out. Therefore, the findings of this study should be verified by large-scale prospective studies and multicenter collaborative studies. Our results should be regarded as merely indicative of the direction for future research. Fifth, differences in experience among surgeons may have affected outcomes. In particular, robotic surgery was performed by a limited number of surgeons, so the results may have depended on the skills of the surgeons. However, we have adjusted for major confounding factors using multivariate analysis, demonstrating that the differences in outcomes between surgical procedures are independent of these factors. Future studies should involve more surgeons in a multicenter collaborative study to examine the effects of surgeons in greater detail.

Robot-assisted CBD surgery appears safe and is associated with faster short-term recovery, particularly in children. Given shorter follow-up and potential confounding, definitive long-term comparative effectiveness requires adjusted, time-to-event analyses and longer observation.

## Supplementary Information

Below is the link to the electronic supplementary material.

Supplementary file1 (PDF 112 KB)

Supplementary file2 (PDF 114 KB)

Supplementary file3 (PDF 159 KB)

Supplementary file4 (PDF 133 KB)

Supplementary file5 (PDF 135 KB)

Supplementary file6 (PDF 133 KB)

Supplementary file7 (PDF 134 KB)

Supplementary file8 (PDF 175 KB)

Supplementary file9 (TIFF 454 KB) Supplementary Fig. 1 Port placement for robot-assisted surgery. (a) Port placement in children An 8-mm 3D camera port (R3) was inserted at the umbilicus, and three 8-mm robotic ports were placed (R1 and R2 on the right lower abdomen and R4 on the left upper abdomen), with a 12-mm assistant port (As) in the left lower abdomen. (b) Port placement in adults A multichannel port was inserted at the umbilicus with an 8-mm 3D camera port (R2) In addition, two 8-mm ports were inserted on either side of the abdomen A 5-mm assistant port was inserted into the left lower abdomen

Supplementary file10 (TIFF 249 KB) Supplementary Fig. 2 Kaplan–Meier curves showing the length of late complication-free survival from CBD surgery. The vertical dashes represent patients who developed late complications. The solid curve represents the patients who underwent laparoscopic surgery, the dashed curve represents those who underwent robot-assisted surgery

Supplementary file11 (TIFF 230 KB) Supplementary Fig. 3 Kaplan–Meier curves showing the length of late complication-free survival from CBD surgery in adults. The vertical dashes represent patients who developed late complications. The solid curve represents the patients who underwent laparoscopic surgery, the dashed curve represents those who underwent robot-assisted surgery

Supplementary file12 (TIFF 2737 KB) Supplementary Fig. 4 Bile ductoplasty in robot-assisted surgery. (a) A membranous stenosis was observed at the right bile duct (within the dotted circle), which was incised in the direction of the arrow using multi-jointed forceps. (b) Using a multi-jointed needle holder, the cut surface was sutured with 6–0 absorbable sutures under 3D magnified vision

## Data Availability

No datasets were generated or analyzed during the current study.
