# Supplementary material for: Safety and efficacy of robot-assisted bile ductoplasty and intrapancreatic bile duct resection in congenital biliary dilatation: a single-center retrospective cohort (2013–2024)
Source: J Robot Surg. 2025 Sep 18;19(1):618. doi: 10.1007/s11701-025-02782-8 (PMC12446100; doi:10.1007/s11701-025-02782-8)
Supplement: Supplementary file 2 — Supplementary file2 (PDF 114 KB) [file 11701_2025_2782_MOESM2_ESM.pdf]

Supplementary Table 6 Characteristics and outcomes excluding non-dilatation type

	Rob (n = 53)	Lap (n = 97)	P value
Male	17 (32%)	25 (26%)	1
Age (year)	8 (2-24)	2 (1-6)	**< 0.001**
<1 year	7 (13%)	21 (22%)	
1–17 years	27 (51%)	66 (68%)	
≥18 years	19 (36%)	10 (10%)	
Body weight (kg)	25 (12-52)	13 (9-21)	**< 0.001**
Todani classification			0.070
1a	14 (26%)	17 (18%)	
1b	1 (1.9%)	0	
1c	12 (23%)	14 (14%)	
IV-A	26 (49%)	66 (68%)	
Perforation	1 (1.9%)	4 (4.1%)	0.66
Operative time (min)	454 (367-556)	410 (351-464)	**0.044**
Blood loss (mL) [a]	24 (15-46)	38 (12-82)	0.12
Bile ductoplasty	28 (53%)	58 (60%)	0.49
Early complication [b]	7 (13%)	8 (8.2%)	0.40
Length of hospital stay (day)	8 (7-11)	10 (8-13)	**< 0.001**
Period until enteral feeding starts (day)	3 (3-4)	4 (3-5)	**< 0.001**
Duration of drain placement (day)	5 (5-7)	6 (6-7)	**0.002**
Late complication (CD ≥I) [c]	2 (3.8%)	18 (19%)	**0.011**
Late complication (CD ≥III) [d]	1 (1.9%)	12 (1.0%)	**0.033**
Residual intrapancreatic bile duct (mm)	0 (0-5)	0 (0-0)	0.077
Cholangiocarcinoma	0	0	

Values are present as n (%) or median (interquartile range). Bold value indicates significant difference.

P values are two-sided.

Rob: Patients underwent robotic-assisted surgery

Lap: Patients underwent laparoscopic surgery

a: Blood loss was measured intraoperatively

b: Complications within 30 days after surgery and Clavien-Dindo classification III or higher

c: Complications 31 days or more after surgery and Clavien-Dindo classification I or higher including events treated conservatively

d: Complications 31 days or more after surgery and Clavien-Dindo classification III or higher including events treated conservatively

Title:

Safety and efficacy of robot-assisted bile ductoplasty and intrapancreatic bile duct resection in congenital biliary dilatation: a single-center retrospective cohort (2013–2024)

Journal:

Journal of Robotic Surgery

Authors:

Daiki Kato, Chiyoe Shirota, Hiroo Uchida, Akinari Hinoki, Satoshi Makita, Katsuhiro Ogawa, Masamune Okamoto, Akihiro Yasui, Shunya Takada, Kaito Hayashi, Yoichi Nakagawa, Hiroki Ishii, Hajime Asai, Hizuru Amano, and Takahisa Tainaka

Affiliation:

Department of Pediatric Surgery, Nagoya University Graduate School of Medicine, 65 Tsurumai-cho, Showa-ku, Nagoya 466-8550, Japan

Correspondence to:

Takahisa Tainaka, MD, PhD

Department of Pediatric Surgery Nagoya University Graduate School of Medicine 65 Tsurumai-cho, Showa-ku, Nagoya 466-8550, Japan

Email: tainaka.takahisa.g2@f.mail.nagoya-u.ac.jp

Tel: +81-52-744-2959 Fax: +81-52-744-2980
