# Supplementary figures and images for: Safety and efficacy of robot-assisted bile ductoplasty and intrapancreatic bile duct resection in congenital biliary dilatation: a single-center retrospective cohort (2013–2024)
Source: J Robot Surg. 2025 Sep 18;19(1):618. doi: 10.1007/s11701-025-02782-8 (PMC12446100; doi:10.1007/s11701-025-02782-8)

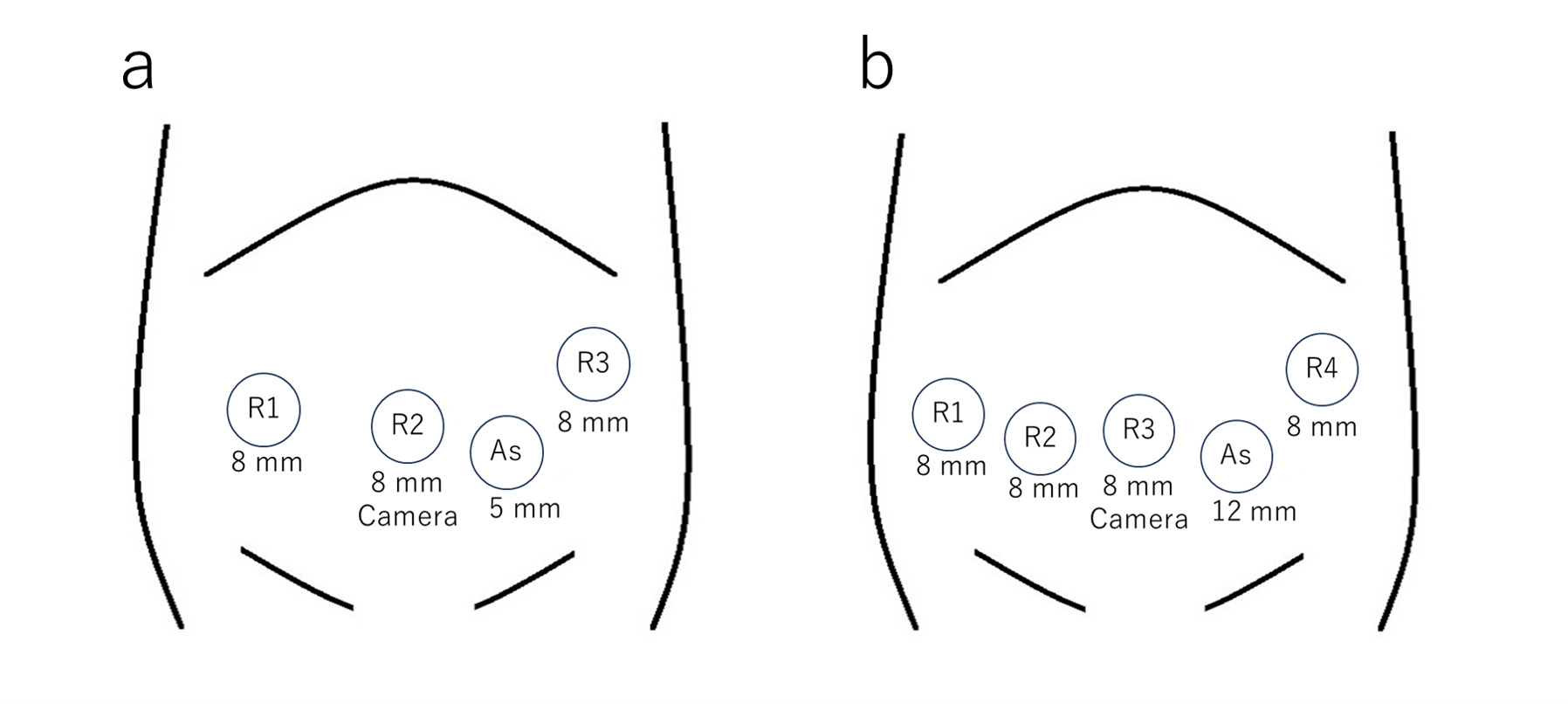

Supplement: Supplementary file 3 — Supplementary file3 (PDF 159 KB) [file 11701_2025_2782_MOESM3_ESM.tiff]

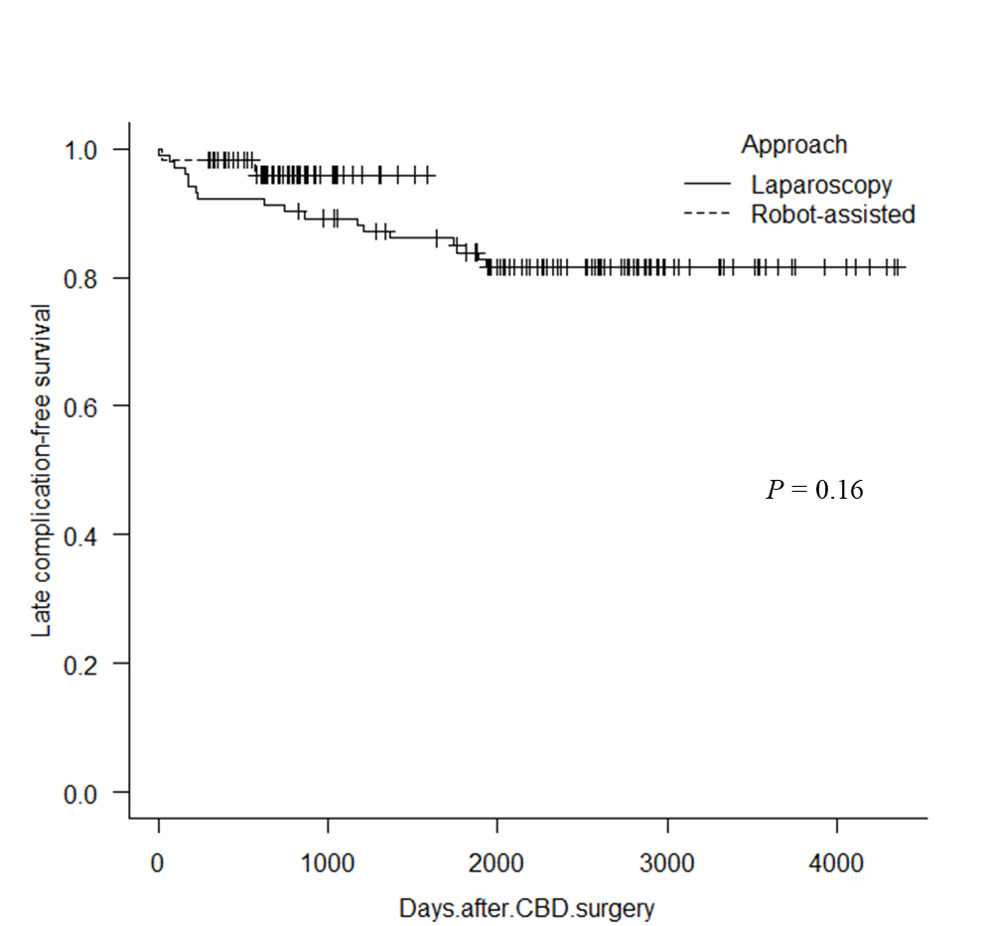

Supplement: Supplementary file 4 — Supplementary file4 (PDF 133 KB) [file 11701_2025_2782_MOESM4_ESM.tiff]

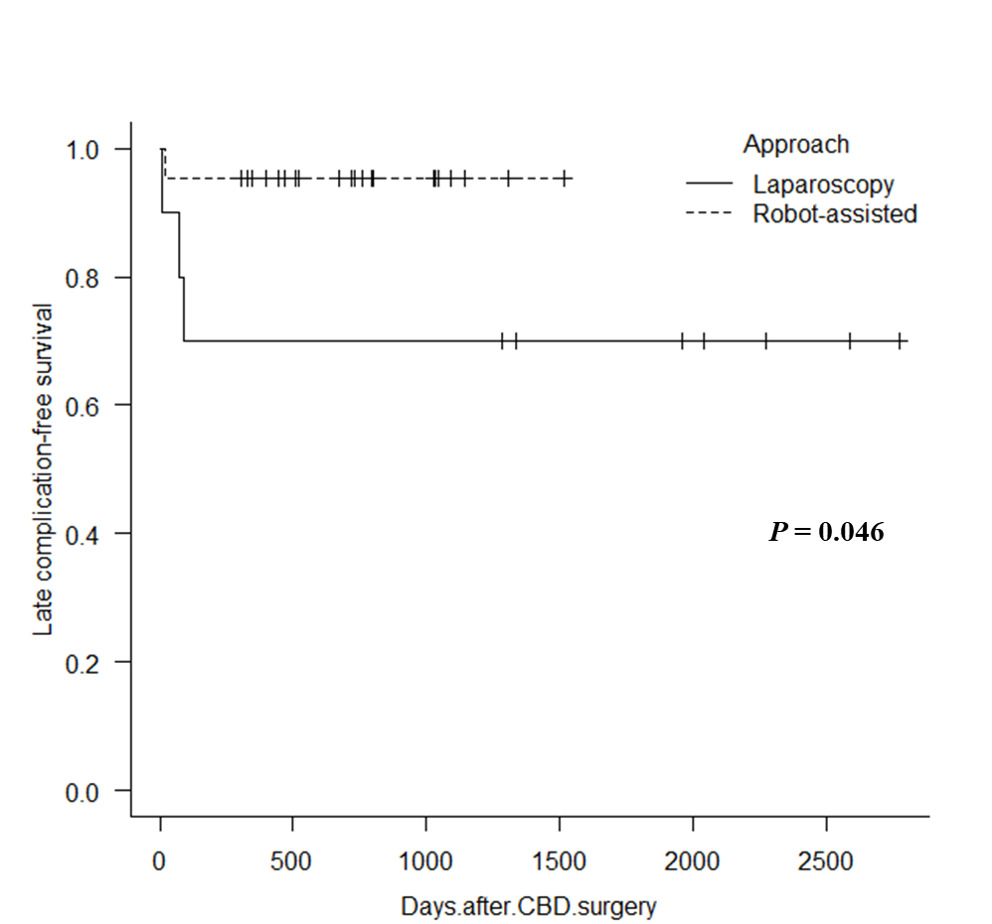

Supplement: Supplementary file 5 — Supplementary file5 (PDF 135 KB) [file 11701_2025_2782_MOESM5_ESM.tiff]

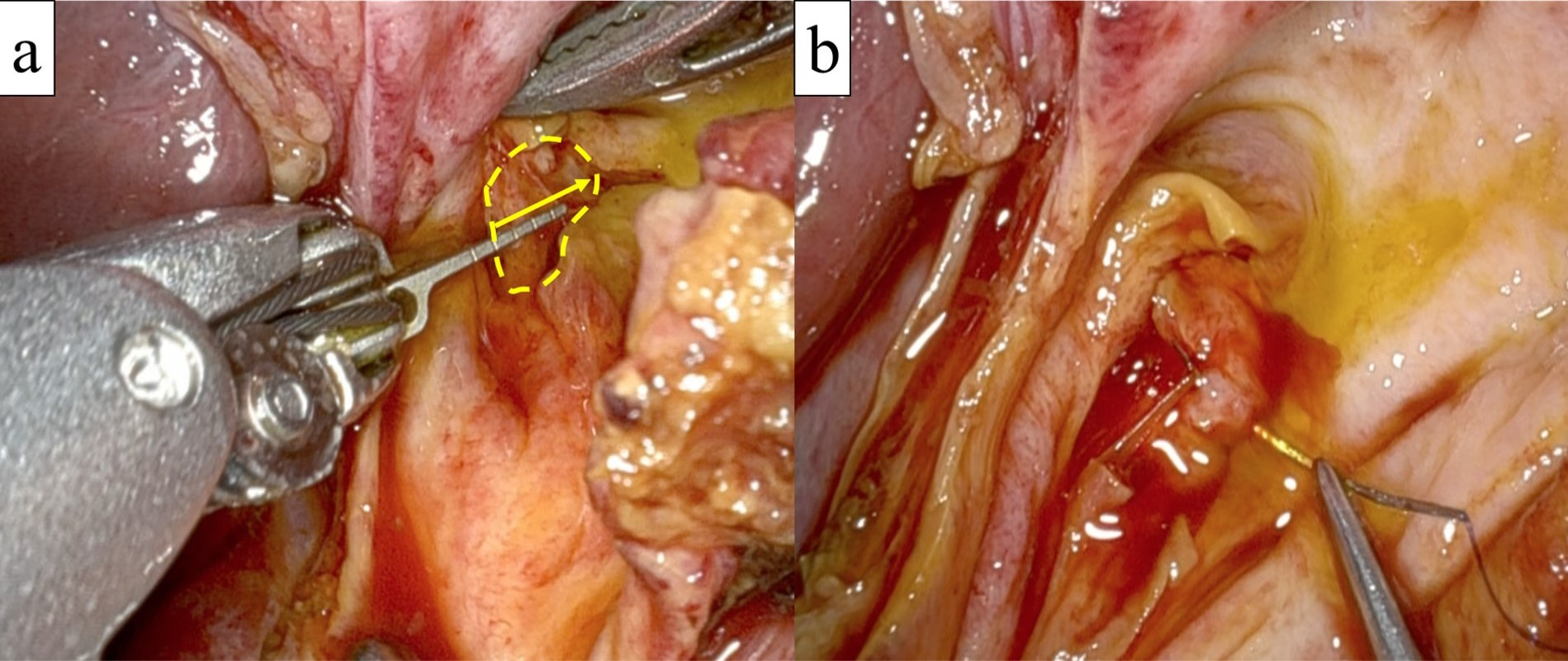

Supplement: Supplementary file 6 — Supplementary file6 (PDF 133 KB) [file 11701_2025_2782_MOESM6_ESM.tiff]
