# Supplementary material for: Safety and efficacy of robot-assisted bile ductoplasty and intrapancreatic bile duct resection in congenital biliary dilatation: a single-center retrospective cohort (2013–2024)
Source: J Robot Surg. 2025 Sep 18;19(1):618. doi: 10.1007/s11701-025-02782-8 (PMC12446100; doi:10.1007/s11701-025-02782-8)
Supplement: Supplementary file 7 — Supplementary file7 (PDF 134 KB) [file 11701_2025_2782_MOESM7_ESM.pdf]

Supplementary Information. 1

The laparoscopic surgical procedures

Laparoscopic surgery was performed with the patients in the supine position using five trocars: two at the umbilicus and one each in the right upper and middle flanks and the left upper abdomen, using an 8–10 mmHg artificial pneumoperitoneum. The common hepatic duct was typically divided 0.5 cm below the bifurcation of the right and left hepatic ducts and 0.5 cm above the junction with the pancreatic duct. The ductal junction was confirmed using intraoperative cholangiography. After creating a Roux-en-Y limb and exteriorizing it through the umbilical incision, the limb was introduced to the hilum via the retrocolic route. An end-to-side hepaticojejunostomy was per formed laparoscopically with a single layer of simple interrupted 5-0 monofilament absorbable sutures.

Title:

Safety and efficacy of robot-assisted bile ductoplasty and intrapancreatic bile duct resection in congenital biliary dilatation: a single-center retrospective cohort (2013–2024)

Journal:

Journal of Robotic Surgery

Authors:

Daiki Kato, Chiyoe Shirota, Hiroo Uchida, Akinari Hinoki, Satoshi Makita, Katsuhiro Ogawa, Masamune Okamoto, Akihiro Yasui, Shunya Takada, Kaito Hayashi, Yoichi Nakagawa, Hiroki Ishii, Hajime Asai, Hizuru Amano, and Takahisa Tainaka

Affiliation:

Department of Pediatric Surgery, Nagoya University Graduate School of Medicine, 65 Tsurumai-cho, Showa-ku, Nagoya 466-8550, Japan

Correspondence to:

Takahisa Tainaka, MD, PhD

Department of Pediatric Surgery Nagoya University Graduate School of Medicine 65 Tsurumai-cho, Showa-ku, Nagoya 466-8550, Japan
Email: tainaka.takahisa.g2@f.mail.nagoya-u.ac.jp
Tel: +81-52-744-2959 Fax: +81-52-744-2980
