# Supplementary material for: Safety and efficacy of robot-assisted bile ductoplasty and intrapancreatic bile duct resection in congenital biliary dilatation: a single-center retrospective cohort (2013–2024)
Source: J Robot Surg. 2025 Sep 18;19(1):618. doi: 10.1007/s11701-025-02782-8 (PMC12446100; doi:10.1007/s11701-025-02782-8)
Supplement: Supplementary file 8 — Supplementary file8 (PDF 175 KB) [file 11701_2025_2782_MOESM8_ESM.pdf]

Supplementary Information. 2

The robot-assisted surgical procedures

Robot-assisted surgery followed the same fundamental surgical procedures, with adjustments in port placement. The da Vinci Surgical System Xi was used with either a four plus one ports or a three plus one ports with a multichannel port. For the five-port method, an 8-mm 3D camera port (R3) was inserted at the umbilicus, and three 8-mm robotic ports were placed (R1 and R2 on the right lower abdomen and R4 on the left upper abdomen), with a 12-mm assistant port (As) in the left lower abdomen. For the four-port method, a multichannel port was inserted at the umbilicus with an 8-mm 3D camera port (R2). In addition, two 8-mm ports were inserted on either side of the abdomen. A 5-mm assistant port was inserted into the left lower abdomen.

Title:

Safety and efficacy of robot-assisted bile ductoplasty and intrapancreatic bile duct resection in congenital biliary dilatation: a single-center retrospective cohort (2013–2024)

Journal:

Journal of Robotic Surgery

Authors:

Daiki Kato, Chiyoe Shirota, Hiroo Uchida, Akinari Hinoki, Satoshi Makita, Katsuhiro Ogawa, Masamune Okamoto, Akihiro Yasui, Shunya Takada, Kaito Hayashi, Yoichi Nakagawa, Hiroki Ishii, Hajime Asai, Hizuru Amano, and Takahisa Tainaka

Affiliation:

Department of Pediatric Surgery, Nagoya University Graduate School of Medicine, 65 Tsurumai-cho, Showa-ku, Nagoya 466-8550, Japan

Correspondence to:

Takahisa Tainaka, MD, PhD

Department of Pediatric Surgery Nagoya University Graduate School of Medicine 65 Tsurumai-cho, Showa-ku, Nagoya 466-8550, Japan
Email: tainaka.takahisa.g2@f.mail.nagoya-u.ac.jp
Tel: +81-52-744-2959 Fax: +81-52-744-2980
