# Supplementary material for: Safety and efficacy of robot-assisted bile ductoplasty and intrapancreatic bile duct resection in congenital biliary dilatation: a single-center retrospective cohort (2013–2024)
Source: J Robot Surg. 2025 Sep 18;19(1):618. doi: 10.1007/s11701-025-02782-8 (PMC12446100; doi:10.1007/s11701-025-02782-8)
Supplement: Supplementary file 9 — Supplementary file9 (TIFF 454 KB) Supplementary Fig. 1 Port placement for robot-assisted surgery. (a) Port placement in children An 8-mm 3D camera port (R3) was inserted at the umbilicus, and three 8-mm robotic ports were placed (R1 and R2 on the right lower abdomen and R4 on the left upper abdomen), with a 12-mm assistant port (As) in the left lower abdomen. (b) Port placement in adults A multichannel port was inserted at the umbilicus with an 8-mm 3D camera port (R2) In addition, two 8-mm ports were inserted on either side of the abdomen A 5-mm assistant port was inserted into the left lower abdomen [file 11701_2025_2782_MOESM9_ESM.pdf]

Supplementary Table 1 Surgical outcomes of the patients with and without bile ductoplasty

	bile ductoplasty + (n = 87)	bile ductoplasty - (n = 72)	P value
Procedure			0.51
Robot-assisted surgery	29 (33%)	28 (39%)	
Laparoscopic surgery	58 (67%)	44 (61%)	
Operative time (min)	438 (364-544)	389 (345-448)	**0.011**
Blood loss (mL) [a]	37 (12-80)	30 (19-62)	0.94
Early complication [b]	7 (12%)	8 (7.8%)	0.59
Bile leakage	5 [c]	4	
Pancreatic fistula	3 [c]	2	
Bowel obstruction	1	1	
Roux-en-Y limb dilatation	0	1	
Hepatolithiasis	4	3	1
Cholangitis	6	7	0.57

Values are present as n (%) or median (interquartile range). Bold value indicates significant difference.

P values are two-sided.

a: Blood loss was measured intraoperatively

b: Complications within 30 days after surgery and Clavien-Dindo classification III or higher

c: The two patients developed bile leakage and pancreatic fistula

Title:

Safety and efficacy of robot-assisted bile ductoplasty and intrapancreatic bile duct resection in congenital biliary dilatation: a single-center retrospective cohort (2013–2024)

Journal:

Journal of Robotic Surgery

Authors:

Daiki Kato, Chiyoe Shirota, Hiroo Uchida, Akinari Hinoki, Satoshi Makita, Katsuhiro Ogawa, Masamune

Okamoto, Akihiro Yasui, Shunya Takada, Kaito Hayashi, Yoichi Nakagawa, Hiroki Ishii, Hajime Asai, Hizuru Amano, and Takahisa Tainaka

Affiliation:

Department of Pediatric Surgery, Nagoya University Graduate School of Medicine, 65 Tsurumai-cho, Showa-ku, Nagoya 466-8550, Japan

Correspondence to:

Takahisa Tainaka, MD, PhD

Department of Pediatric Surgery Nagoya University Graduate School of Medicine 65 Tsurumai-cho, Showa-ku, Nagoya 466-8550, Japan

Email: tainaka.takahisa.g2@f.mail.nagoya-u.ac.jp

Tel: +81-52-744-2959 Fax: +81-52-744-2980
