# Supplementary material for: Safety and efficacy of robot-assisted bile ductoplasty and intrapancreatic bile duct resection in congenital biliary dilatation: a single-center retrospective cohort (2013–2024)
Source: J Robot Surg. 2025 Sep 18;19(1):618. doi: 10.1007/s11701-025-02782-8 (PMC12446100; doi:10.1007/s11701-025-02782-8)
Supplement: Supplementary file 11 — Supplementary file11 (TIFF 230 KB) Supplementary Fig. 3 Kaplan–Meier curves showing the length of late complication-free survival from CBD surgery in adults. The vertical dashes represent patients who developed late complications. The solid curve represents the patients who underwent laparoscopic surgery, the dashed curve represents those who underwent robot-assisted surgery [file 11701_2025_2782_MOESM11_ESM.pdf]

Supplementary Table 3 Cox regression analysis for predictors of length of hospital stay

	HR	95% CI	P value
Age (Y)	1.000	0.976 - 1.022	0.88
Body weight (kg)	0.991	0.974 - 1.007	0.27
Todani classification type IV-A	1.000	0.685 - 1.373	0.86
Perforation	0.841	0.333 - 2.123	0.71
Approach (robot-assisted)	**1.680**	**1.154 - 2.446**	**0.007**
Bile ductoplasty	0.914	0.666 - 1.256	0.58

Bold value indicates significant difference.

P values are two-sided.

HR: Hazard ratio

CI: Confidence interval

Title:

Safety and efficacy of robot-assisted bile ductoplasty and intrapancreatic bile duct resection in congenital biliary dilatation: a single-center retrospective cohort (2013–2024)

Journal:

Journal of Robotic Surgery

Authors:

Daiki Kato, Chiyoe Shirota, Hiroo Uchida, Akinari Hinoki, Satoshi Makita, Katsuhiro Ogawa, Masamune Okamoto, Akihiro Yasui, Shunya Takada, Kaito Hayashi, Yoichi Nakagawa, Hiroki Ishii, Hajime Asai, Hizuru Amano, and Takahisa Tainaka

Affiliation:

Department of Pediatric Surgery, Nagoya University Graduate School of Medicine, 65 Tsurumai-cho, Showa-ku, Nagoya 466-8550, Japan

Correspondence to:

Takahisa Tainaka, MD, PhD

Department of Pediatric Surgery Nagoya University Graduate School of Medicine 65 Tsurumai-cho, Showa-ku, Nagoya 466-8550, Japan

Email: tainaka.takahisa.g2@f.mail.nagoya-u.ac.jp

Tel: +81-52-744-2959 Fax: +81-52-744-2980
