# Supplementary material for: Safety and efficacy of robot-assisted bile ductoplasty and intrapancreatic bile duct resection in congenital biliary dilatation: a single-center retrospective cohort (2013–2024)
Source: J Robot Surg. 2025 Sep 18;19(1):618. doi: 10.1007/s11701-025-02782-8 (PMC12446100; doi:10.1007/s11701-025-02782-8)
Supplement: Supplementary file 12 — Supplementary file12 (TIFF 2737 KB) Supplementary Fig. 4 Bile ductoplasty in robot-assisted surgery. (a) A membranous stenosis was observed at the right bile duct (within the dotted circle), which was incised in the direction of the arrow using multi-jointed forceps. (b) Using a multi-jointed needle holder, the cut surface was sutured with 6–0 absorbable sutures under 3D magnified vision [file 11701_2025_2782_MOESM12_ESM.pdf]

Supplementary Table 4 Cox regression analysis for predictors of time to enteral feeding

	HR	95% CI	P value
Age (Y)	1.000	0.978 - 1.024	0.95
Body weight (kg)	0.991	0.976 - 1.007	0.26
Todani classification type IV-A	0.979	0.695 - 1.379	0.90
Perforation	0.999	0.400 - 2.454	1
Approach (robot-assisted)	**1.602**	**1.106 - 2.322**	**0.013**
Bile ductoplasty	0.762	0.550 - 1.057	0.10

Bold value indicates significant difference.

P values are two-sided.

HR: Hazard ratio

CI: Confidence interval

Title:

Safety and efficacy of robot-assisted bile ductoplasty and intrapancreatic bile duct resection in congenital biliary dilatation: a single-center retrospective cohort (2013–2024)

Journal:

Journal of Robotic Surgery

Authors:

Daiki Kato, Chiyoe Shirota, Hiroo Uchida, Akinari Hinoki, Satoshi Makita, Katsuhiro Ogawa, Masamune Okamoto, Akihiro Yasui, Shunya Takada, Kaito Hayashi, Yoichi Nakagawa, Hiroki Ishii, Hajime Asai, Hizuru Amano, and Takahisa Tainaka

Affiliation:

Department of Pediatric Surgery, Nagoya University Graduate School of Medicine, 65 Tsurumai-cho, Showa-ku, Nagoya 466-8550, Japan

Correspondence to:

Takahisa Tainaka, MD, PhD

Department of Pediatric Surgery Nagoya University Graduate School of Medicine 65 Tsurumai-cho, Showa-ku, Nagoya 466-8550, Japan

Email: tainaka.takahisa.g2@f.mail.nagoya-u.ac.jp

Tel: +81-52-744-2959 Fax: +81-52-744-2980
